# RAB25 modulates pit cell commitment by coordinating transforming growth factor-alpha secretion from gastric epithelial cells

**DOI:** 10.1038/s41419-025-08316-2

**Published:** 2025-12-09

**Authors:** Haengdueng Jeong, Yura Lee, Chanyang Uhm, Seok Young Hwang, Sumin Hur, Minsoo Noh, Robert J. Coffey, James R. Goldenring, Ki Taek Nam

**Affiliations:** 1 Department of Biomedical Sciences, Brain Korea 21 PLUS Project for Medical Science, Yonsei University College of Medicine, Seoul, Korea; 2 Epithelial Biology Center and Department of Surgery, Vanderbilt University Medical Center and the Nashville VA Medical Center, Nashville, TN USA; 3 Department of Molecular and Life Science, Hanyang University, Ansan, Republic of Korea; 4 Natural Products Research Institute, College of Pharmacy, Seoul National University, Seoul, Korea; 5 Epithelial Biology Center and Department of Medicine, Vanderbilt University School of Medicine, Nashville, TN USA

**Keywords:** Protein translocation, Differentiation, Stomach diseases

## Abstract

EGFR signaling serves as a regulator of lineage commitment in the stomach. A recent study revealed that two different EGFR ligands can induce fate determination of isthmus progenitors in corpus, but the source and regulatory mechanism of the ligands remain unclear. We analyzed single-cell RNA sequencing and found that Rab25 was strongly expressed in epithelial cells in upper corpus glands along with transforming growth factor-alpha (TGFA) associated with pit lineage commitment. Using mouse primary cell culture, we found that Rab25 loss facilitated TGFA secretion and subsequently promoted upregulation of EGFR signaling in the pit region. Long-term alteration of TGFA secretion in Rab25 KO mice caused gastric lesions with massive foveolar hyperplasia. Most importantly, this corpus lesion was ameliorated by neutralization of TGFA. Moreover, *RAB25* expression was reduced in human Ménétrier’s disease. Collectively, we provide evidence for a physiological role of Rab25 in the gastric environment to maintain normal lineage commitment.

## Introduction

The stomach is lined with a mucosa containing glandular structures harboring functional epithelial cells. In the stomach corpus gland of the mouse, proliferating progenitor cells are located in the upper gland region referred to as the isthmus. In homeostasis, the isthmus progenitor cells produce secondary lineage-committed progenitors in which the molecular signatures of progenitor and matured cells transiently coexist [1, 2]. The secondary lineage-committed progenitors give rise to mature cell lineages. Notably, the secondary progenitor cells give rise to parietal cells or chief cells migrating towards the bases that can survive for several weeks or months, whereas the cells that migrate upward differentiate into pit cells and survive only for a few days [3–5]. The distinct epithelial cells in the corpus play unique roles and, at the end of their lifespan, the cells are constantly replaced by isthmus cell division and differentiation.

To maintain this organized process, normal physiological events involving the secretion of gastric acid, hormones, growth factors, and neurotransmitters are pivotal [6, 7]. Unexpected lineage alteration and gastric lesions may occur due to distorted physiological conditions. Indeed, achlorhydria resulting from dysfunction of parietal cells induces hyperplasia as well as mucous cell metaplasia [8]. Hypergastrinemia, characterized by an increase in serum gastrin, causes acid hypersecretion, foveolar hyperplasia, and enterochromaffin-like cell (ECL) hyperplasia [9, 10]. Additionally, upregulation or downregulation of growth factors promotes spontaneous gastric lesions in mice [11–13]. Because of these characteristics, physiological mechanisms must be controlled precisely.

It appears that diverse epidermal growth factor receptor (EGFR) ligands influence corpus homeostasis. Recently, Takada et al. [1] found that transforming growth factor alpha (TGFA) can promote progenitor differentiation into pit cells by activating EGFR signaling. Also, our recent investigation demonstrated that amphiregulin and TGFA can switch the differentiation fate of progenitor cells. [13] TGFA and amphiregulin directed different lineage commitments to pit cells and parietal cells, respectively. In humans, S Wölffling et al. [14] suggested that the concentration of EGF and BMP determines the lineage patterning. TGFA, one of the crucial growth factors, is initially expressed as pro-TGFA, transported to the membrane through vesicle trafficking, and cleaved by proteases for extracellular secretion. It shares 35% homology with EGF and has a nearly identical spectrum of biological activity, activating EGFR downstream signaling. TGFA is a highly conserved growth factor in a broad range of species, suggesting that it plays a critical role in the gastric mucosa [15]. Overexpression of TGFA causes an alteration of gastric lineage cell commitment towards pit cells, and aberrant TGFA/EGFR signaling is also associated with the development of a rare hypertrophic disorder, Ménétrier’s disease, characterized by massive foveolar hyperplasia [12, 16, 17]. Indeed, blockade of EGFR ameliorated Ménétrier’s disease along with reduction of foveolar hyperplasia [18]. Nevertheless, there is a lack of evidence for how expression of TGFA is regulated in gastric epithelial cells.

Rab proteins are small GTP-binding proteins that serve as key regulators of intracellular trafficking [19]. They reside in distinct membrane compartments and coordinate critical processes such as vesicle endocytosis, exocytosis, and recycling [19]. Several reports have demonstrated that Rab11 family members (Rab11a, Rab11b, and Rab25) are expressed in gastric cells [20–24]. Rab25 plays epithelial-specific roles in multiple organs with diverse targets [25–28]. However, in the stomach, a molecular function has been defined only for Rab11a, which regulates fusion of tubulovesicles with the secretory canaliculus in acid-secreting parietal cells [21, 29]. Here, we elucidated a physiological role for Rab25 in the coordination of TGFA secretion in gastric epithelial cells. Rab25 acted as a regulator of TGFA secretion, and loss of RAB25 promoted pit cell commitment and eventually induced gastric lesions through long-term TGFA/EGFR activation. Most importantly, neutralization of TGFA in Rab25 knock-out (KO) mice ameliorated the gastric lesions. Our investigation suggests that TGFA secretion is modulated by a Rab25-dependent mechanism.

## Results

### **Rab25 expression is concentrated in epithelial cells of the upper corpus in both human and mice**

**Fig. 1:**
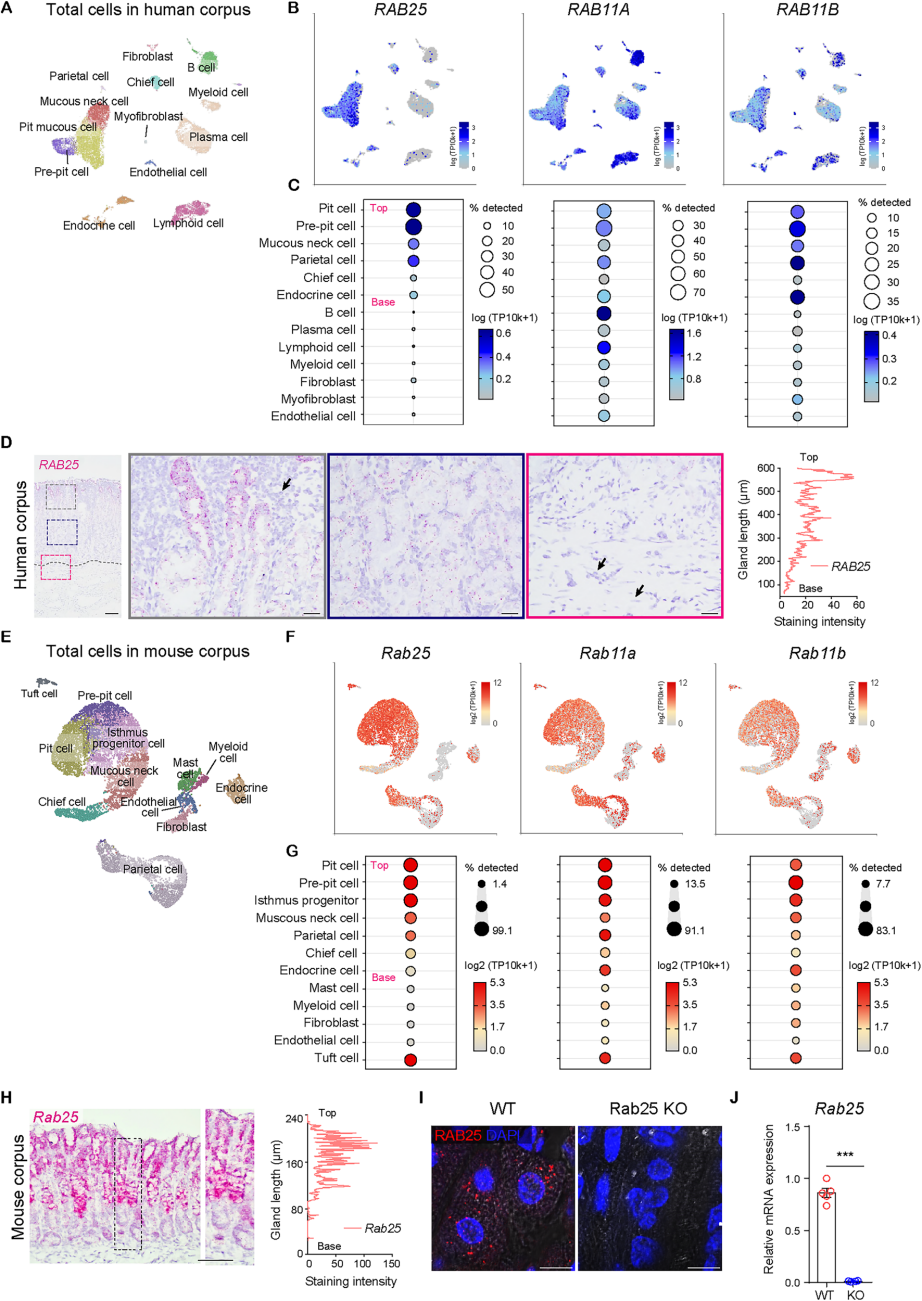
*RAB25*- and *Rab25*-expressing cell types are determined by staining and single-cell profiling. **A** Uniform manifold approximation and projection (UMAP) of 17,083 cells in 13 color-coded clusters. **B**, **C** Bubble plot showing variable expression of human *RAB25, RAB11A, RAB11B* in defined clusters and UMAP showing the distribution of *RAB11* family*-*expressing cells in total cell clusters. Epithelial cells were arranged according to their location (from ‘base’ to ‘top’) within the gland. **D** In situ hybridization images for *RAB25* in the stomach of inflamed normal patient. The black arrows indicate non-epithelial cells (immune cell, fibroblast, and endothelial cell) in lamina propria and submucosa of corpus gland. Scale bars, 100 μm (left panel); 20 μm (enlarged panels). Staining intensity (represented as arbitrary unit, a.u.) was measured according to gland length. **E** UMAP of 12,699 cells in 12 color-coded clusters. **F**, **G** Bubble plot showing variable expression of mouse *Rab25, Rab11a and Rab11b* in defined clusters and UMAP showing the distribution of *Rab11* family*-*expressing cells in total cell clusters. Epithelial cells were arranged according to their location (from ‘base’ to ‘top’) within the gland. **H** In situ hybridization images for *RAB25* in wild-type (WT) corpus. Scale bars, 100 μm. Staining intensity (represented as arbitrary unit, a.u.) was measured according to gland length. **I**, **J** Immunofluorescence images and RT-qPCR for RAB25/*Rab25* in corpus of WT and Rab25 knock-out (KO) mice (*n* = 4 per group, two tailed Student’s *t*-test, ***P < 0.001). Scale bars, 50 μm. All data are represented as mean ± SEM.

To identify subsets that express RAB11 family, including *RAB25*, we performed single-cell RNA sequencing (scRNA-seq) using the data previously performed by our group [30]. We analyzed and re-clustered a total of 17,083 cells from three inflamed normal gastric mucosa samples that were derived from adjacent non-cancerous sites, and then acquired 16 different subpopulations through unsupervised clustering (Figs. 1A and S1A). The cell types were characterized as Pit cell (*MUC5AC*, *TFF1*), Pre-pit cell (*MUC5AC*, *UBE2C*, *TOP2A*), Mucous neck cell (*MUC6*), Chief cell (*PGA4*, *PGA5*), Parietal cell (*ATP4A*, *ATP4B*, *CBLIF*), Endocrine cell (*CHGA*, *CHGB*), B cell (*MS4A1*, *BANK1*), Plasma cell (*JCHAIN*), Lymphoid cell (*CD3D*, *CD3E*), Myeloid cell (*TREM1*, *S100A9*), Fibroblast (*LUM*, *PDPN*, *POSTN*), Myofibroblast (*ACTA2*), and Endothelial cell (*VWF*, *TPO*, *CD34*), based on known marker genes of various cell types (Fig. S1B, C). Unlike other Rab11 family members, which displayed universal expression across epithelial and non-epithelial clusters, *RAB25* expression was prominent in epithelial cells in the corpus of the human stomach (Fig. 1B, C). In particular, strong expression of *RAB25* was observed in upper regions of corpus glands, and there were no *RAB25* transcripts in cells in the submucosa and lamina propria (Fig. 1B, D). Non-epithelial clusters rarely produce *RAB25* (Fig. 1B, C). scRNA-seq showed that a broad range of epithelial clusters have *RAB25*, and the highest expression was shown in pit lineage cells (Fig. 1B, C). Compared to pit and parietal cells, there was weak expression of *RAB25* in the other epithelial clusters present in the base of the gastric gland (Fig. 1B-D).

To examine *Rab25* distribution in mice, we analyzed and re-clustered a total of 12,699 cells from normal corpus samples, and then acquired 12 different subpopulations through unsupervised clustering using a published data [31] (Figs. 1E, S2A). The cell types were characterized as Pit cell (*Muc5AC*, *Tff1*), Pre-pit cell (*Muc5AC*, *Tff1*, Cell cycle-related genes), Isthmus progenitor (*Top2a*, *Mki67*, *Hmgb2*, *Stmn1*), mucous neck cell (*Muc6*, *Gkn3*), Chief cell (*Chia1*, *Bhlha15*), Parietal cell (*Atp4a*, *Atp4b*, *Apoa1*), Enteroendocrine cell (*Chga*, *Chgb*), Mast cell (*Cma1*, *Cpa3*), Myeloid cell (*Ptprc*, *Lyz2*), Fibroblast (*Col1a2*, *Col3a1*), Endothelial cells (*Pecam1*, *Fabp4*), and Tuft cell (*Dclk1*, *Trpm5*), based on known marker genes of various cell types (Fig. S2B, C). Similar to human, *Rab25* expression was enriched in epithelial cells in upper corpus, involving pit lineage cells, progenitor cells, and some mucous neck cells and parietal cells (Fig. 1F, G). Unlike *Rab11a* and *Rab11b*, *Rab25* was rare in non-epithelial clusters (Fig. 1F, G). In situ hybridization clearly demonstrated that *Rab25 transcripts* were predominantly expressed in the upper regions of corpus glands, where progenitor cells, mucous neck cells, parietal cells, and pit cells reside (Fig. 1H). Indeed, patchy protein expression of RAB25 was evident in epithelial cells in WT mice, but expression was lost in those cells in Rab25 KO mice (Fig. 1I, J). These findings suggested that Rab25 may have a putative function in certain populations of gastric epithelial cells.

### Development of spontaneous gastric lesions in aged Rab25 KO mice

**Fig. 2:**
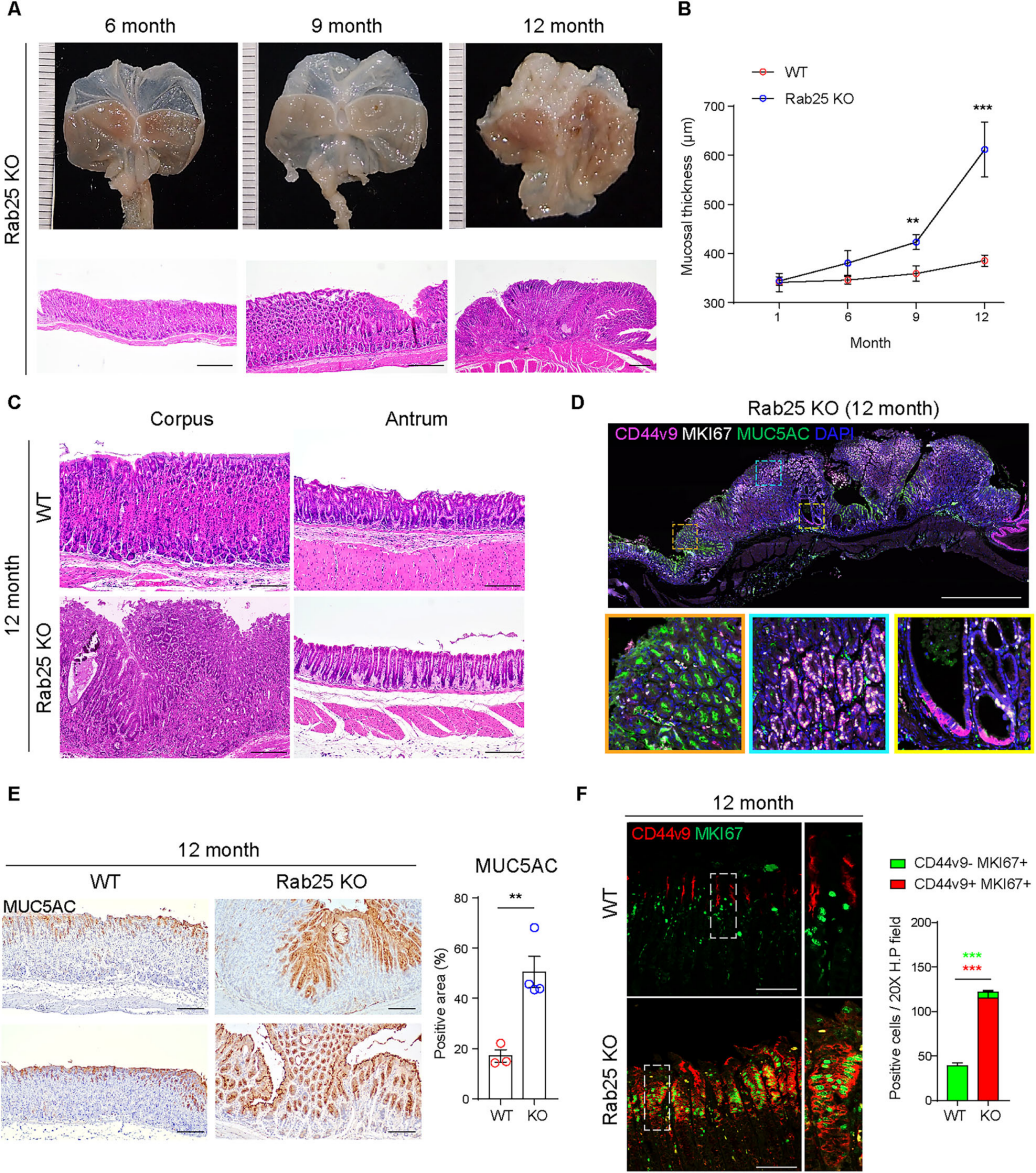
Rab25 loss spontaneously induced massive hyperplasia and transition of epithelial cells. **A** Macroscopic and histopathological observation of 6-, 9-. 12-month-old Rab25 knock-out (KO) mice. Scale bars, 500 μm. **B** The graph represents the mucosal thickness of the corpus in wild-type (WT) and Rab25 KO mice (*n* = 3–4 per group, two-tailed Student’s *t*-test, **P < 0.01 ***P < 0.001). **C** Representative H&E images of the corpus and antrum of 12-month-old WT and Rab25 KO mice. Scale bars, 200 μm. **D** Immunofluorescence images for CD44v9, MUC5AC, and MKI67 in 12-month-old Rab25 KO mice. Scale bar, 1 mm. **E** Immunohistochemistry images for MUC5AC in 12-month-old WT and Rab25 KO mice. The graph indicates MUC5AC positive area in a 10X high-power field (*n* = 3–4 per group, two-tailed Student’s *t*-test, **P < 0.01). Scale bars, 200 μm. **F** Immunofluorescence images for CD44v9 and MKI67 in the corpus of 12-month-old WT and Rab25 KO mice. The graph indicates the number of CD44v9-/MKI67+ and CD44v9+/MKI67+ cells in a 20X high-power field (*n* = 3–4 per group, two tailed Student’s *t*-test, ***P < 0.001). Apical mucin staining for CD44v9 is artifactual. Scale bars, 100 μm. All data are represented as mean ± SEM.

We sought to explore whether loss of Rab25 impacts gastric tissues. To investigate this hypothesis, WT and Rab25 KO mice were sacrificed at 6-, 9-, 12-months of age and histopathological changes were examined. Before 6 months, there were no obvious differences in histological features between the two groups (Fig. 2A, B). However, after 9 months, a significant increase in mucosal thickness was identified in Rab25 KO mice, especially in the pit region (Fig. 2A, B). Subsequently, enlarged gastric rugal folds containing massive hyperplasia were macroscopically observed in 12-month-old Rab25 KO mice compared to WT mice (Figs. 2A, B, S3A). Histopathological observation showed the presence of hyperplastic lesions accompanied by cystic dilatation in the corpus of aged Rab25 KO mice (Fig. 2A–C). This pathology in Rab25 KO mice was not identified in aged WT mice (Figs. 2C and S3A). Unlike corpus, there were no pathological changes in the antrum in both aged WT and Rab25 KO mice (Fig. 2C).

All corpus glands in aged mice did not share the same molecular characteristics and pathology. Immunostaining clearly showed that the corpus in aged Rab25 KO mice harbored two distinct regions, one highly proliferative and the other highly differentiated and less proliferative (Figs. 2D and S3B). To verify the differentiated zone, we stained for MUC5AC, a marker for pit lineage cells, and found that the pit region was markedly increased in aged Rab25 KO mice compared to aged WT mice (Fig. 2E). To characterize the aberrant proliferating cells in corpus, we stained for CD44v9 (CD44 variant 9), which is a marker of metaplasia or stress expressed in basolateral membrane [32, 33]. Numerous proliferating CD44v9+ cells were exclusively present in aged Rab25 KO mice, and those pit cells rarely positive for differentiation marker (Fig. 2D and F). Collectively, Rab25-deficiency resulted in long-term foveolar hyperplasia in the gastric corpus gland, but did not cause gastric neoplasia.

### Pit cells are the primary lineage affected by Rab25 deficiency

**Fig. 3:**
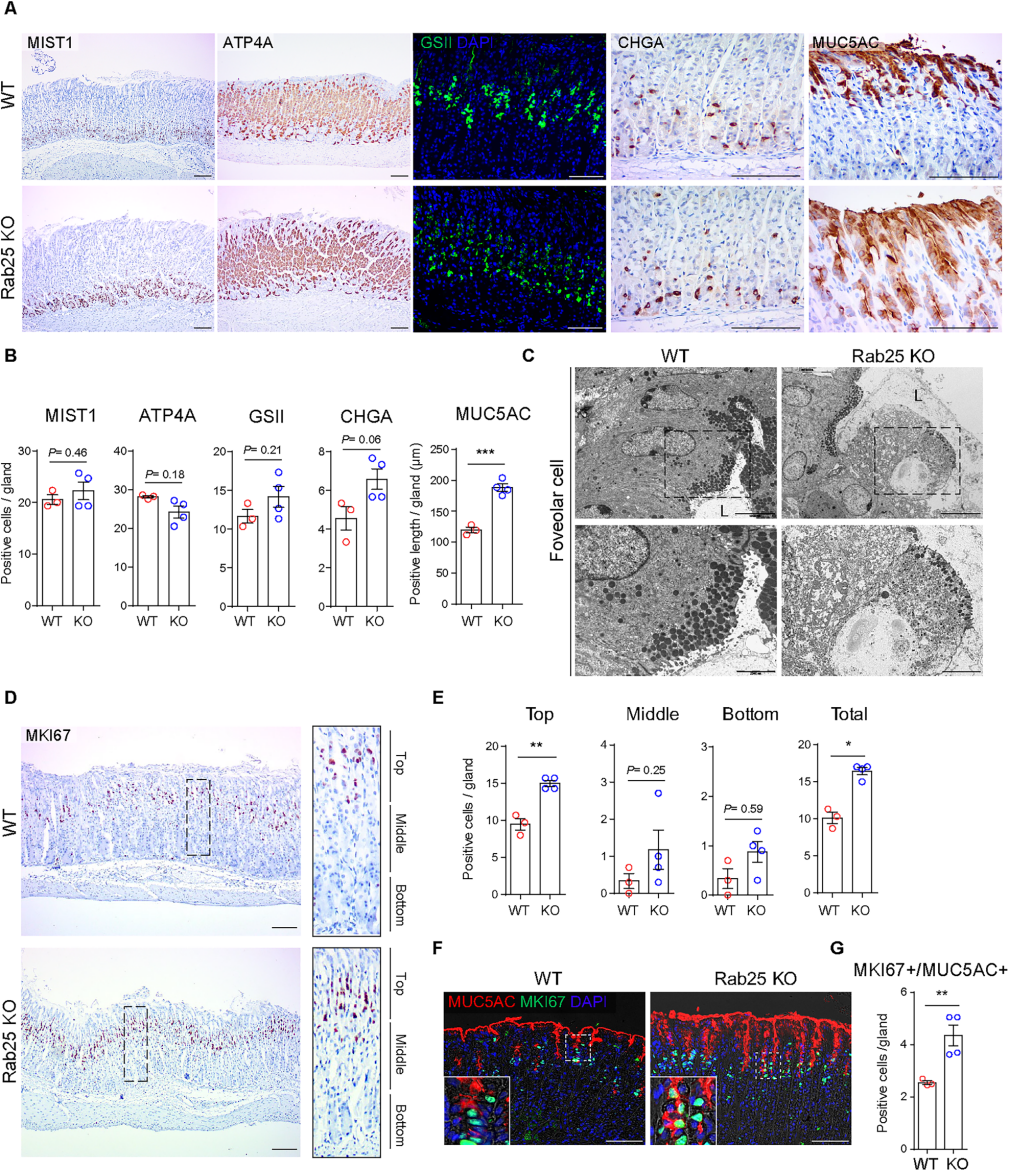
Assessment of corpus lineages in young Rab25 knock-out (KO) mice. **A** Immunohistochemistry and immunofluorescence images for MIST1, ATP4A, GSII, CHGA, MUC5AC. Scale bars, 100 μm. **B** The graphs represent the number of positive cells (MIST1, ATP4A, GSII, and CHGA) and positive length (MUC5AC) per single corpus gland (*n* = 3–4 per group, two-tailed Student’s *t*-test, ***P < 0.001). **C** Transmission electron microscopy images of pit cells in 1-month-old wild-type (WT) and Rab25 KO mice. Scale bars, 5000 nm (upper panels); 2000 nm (lower panels). ‘L’ indicates the gastric lumen. **D** Immunohistochemistry images for MKI67 in 1-month-old WT and Rab25 KO mice. Enlarged panels show the distribution of proliferating cells according to gland length. Scale bars, 100 μm. **E** MKI67+ proliferating cells in single glands were quantified in WT and Rab25 KO mice. Cell numbers were measured at the top, middle, and bottom, each representing one-third of the corpus gland length (*n* = 3–4 per group, two-tailed Student’s *t*-test, *P < 0.05, **P < 0.01). **F** Immunofluorescence images for MUC5AC and MKI67 in the corpus of 1-month-old WT and Rab25 KO mice. Scale bars, 100 μm. **G** The graph represents the number of MKI67 + /MUC5AC+ cells in a corpus gland (*n* = 3–4 per group, two-tailed Student’s *t*-test, **P < 0.01). All data are represented as mean ± SEM.

Since the foveolar hyperplastic regions were patchy in aged Rab25 KO mice, we evaluated 1-month-old WT and Rab25 KO mice to assess early changes resulting from the absence of Rab25. We first examined changes in corpus lineages using representative markers: MIST1 (a transcription factor, also known as BHLHA15) positive chief cells, ATP4A (H+/K+-ATPase transporter subunit) positive parietal cells, GSII (Lectin from *Griffonia Simplicifolia* II) positive mucous neck cells, CHGA (chromogranin A) positive ECL cells, and MUC5AC (the oligomeric mucus/gel-forming protein) positive pit cells. Chief cells, parietal cells, mucous neck cells, and ECL cells were slightly changed or unchanged, and the numbers did not significantly differ in Rab25 KO mice compared to WT mice (Fig. 3A, B). Of note, gastrin-secreting G cells present in antrum also did not change between the two groups (Fig. S3C, D). However, unlike other corpus cell lineages, MUC5AC+ pit cell lineages were markedly increased in Rab25 KO mice compared to WT mice (Fig. 3A, B). In addition, ultrastructure images revealed the presence of abnormal pit cells characterized by irregular cellular morphology and large vacuoles in the cytoplasm (Fig. 3C).

In the mouse corpus, proliferating isthmus progenitor cells can contribute to gland homeostasis by generating secondary lineage-committed progenitors [2]. Also, these secondary lineage-committed progenitors appear to temporally share molecular signatures of both progenitor cells and mature cell lineages [1, 2]. Compared to WT mice, Rab25 KO mice showed an elevation of total MKI67+ proliferating cells in corpus (Fig. 3D, E). In particular, the increase in labeling pattern was evident in the top region of corpus glands, where isthmus progenitors and their lineage-committed daughter cells exist (Fig. 3D, E). Indeed, dual MKI67/MUC5AC positive pre-pit cells were increased in Rab25 KO mice compared to WT mice (Fig. 3F, G). However, there were no marked changes in other corpus lineage-committed progenitor cells. The number of MKI67 + /lineage (parietal cell, mucous neck cell, chief cell, ECL cell) marker+ cells were extremely low or absent (Fig. S4A–D). Collectively, observation of young mice suggests that Rab25 function is linked to pit cell commitment.

### Rab25 is not involved in the physiological function of gastric acid secretion

**Fig. 4:**
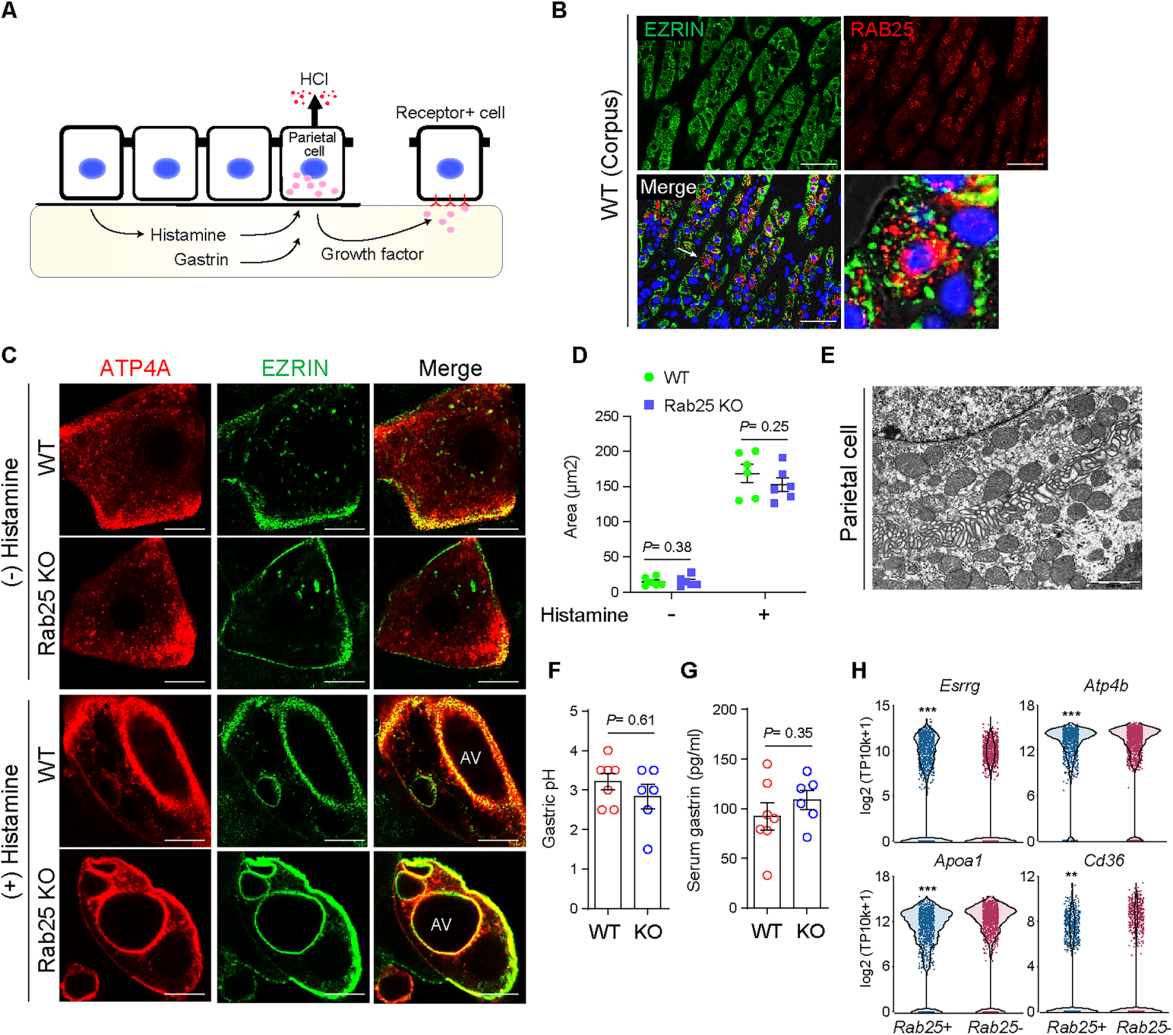
Rab25 deficiency did not affect the acid secretion function of parietal cells. **A** Schematic image shows the physiological role of parietal cells. Parietal cells can secrete gastric acid (HCl) upon hormone stimulation or can produce EGF-ligand. **B** Immunofluorescence images for EZRIN and RAB25 in the corpus of wild-type (WT) mice. Scale bars, 100 μm. **C** Immunocytochemistry images for ATP4A and EZRIN in the cultured parietal cells. Apical vacuole (AV) formation was expanded by histamine treatment for 30 minutes. Scale bars, 10 μm. **D** The graph represents the total area of AV (*n* = 6 per group, ANOVA multiple comparisons test). **E** Transmission electron microscopy images of parietal cells in Rab25 knock-out (KO) mice. Scale bar, 2000 nm. **F**, **G** Measurement on gastric pH and serum gastrin in WT and Rab25 KO mice (*n* = 6–7 per group). **H** The graphs represent the normalized expression levels of selected genes exclusively expressed in parietal cells (*n* = 937 and 1222 for each group). The significance was obtained using Partek Flow’s Hurdle model (**P < 0.01, ***P < 0.001). All data are represented as mean ± SEM.

Based on the distribution of Rab25 and previous results for its related family members, Rab25 may contribute to the parietal cell’s multiple physiological roles such as gastric acid secretion and growth factor production, which are crucial for maintaining corpus homeostasis (Fig. 4A). Indeed, RAB11A was previously identified as a regulator of fusion of proton pump-containing tubulovesicles with the secretory canaliculus in parietal cells [20, 29]. However, punctate RAB25 staining was not colocalized with canaliculus markers in parietal cells (Fig. 4B).

To determine whether loss of Rab25 leads to abnormal acid secretion in parietal cells, primary cultured parietal cells from WT and Rab25 KO mice were stimulated by histamine. In vitro parietal cells showed the redistribution of H/K-ATPase-rich tubulovesicles and formed large intracellular canalicular vacuoles upon stimulation, and parietal cells from WT and Rab25 KO mice responded normally to histamine (Fig. 4C, D). Rab25 KO mice also exhibited expanded intracellular canaliculi shown in stimulated parietal cells in an ultrastructure image (Fig. 4E). As a result, there were no changes in gastric pH and serum gastrin between WT and Rab25 KO mice (Fig. 4F-G). Consistently, the aged mice also exhibited no changes in gastric pH and serum gastrin (Fig. S5A, B). These physiological characteristics of Rab25 KO mice suggested that the foveolar hyperplasia was not associated with achlorhydria and hypergastrinemia and that other mechanisms might be involved. Additionally, we divided the total cells in the parietal cell cluster into *Rab25*+ and *Rab25*- populations (Figs. 1E, F, S5C) and found that *Rab25*+ parietal cells exhibit less mature characteristics compared to their counterpart. *Rab25*+ parietal cells have relatively low expression levels of *Esrrg*, *Atp4b*, *Apoa1*, and *Cd36* (Fig. 4H), which are critical for parietal cell differentiation and maturation and show the highest expression levels in fully mature cells [2, 34].

### Rab25 can coordinate TGFA secretion and its impact on EGFR activation in pit cells

**Fig. 5:**
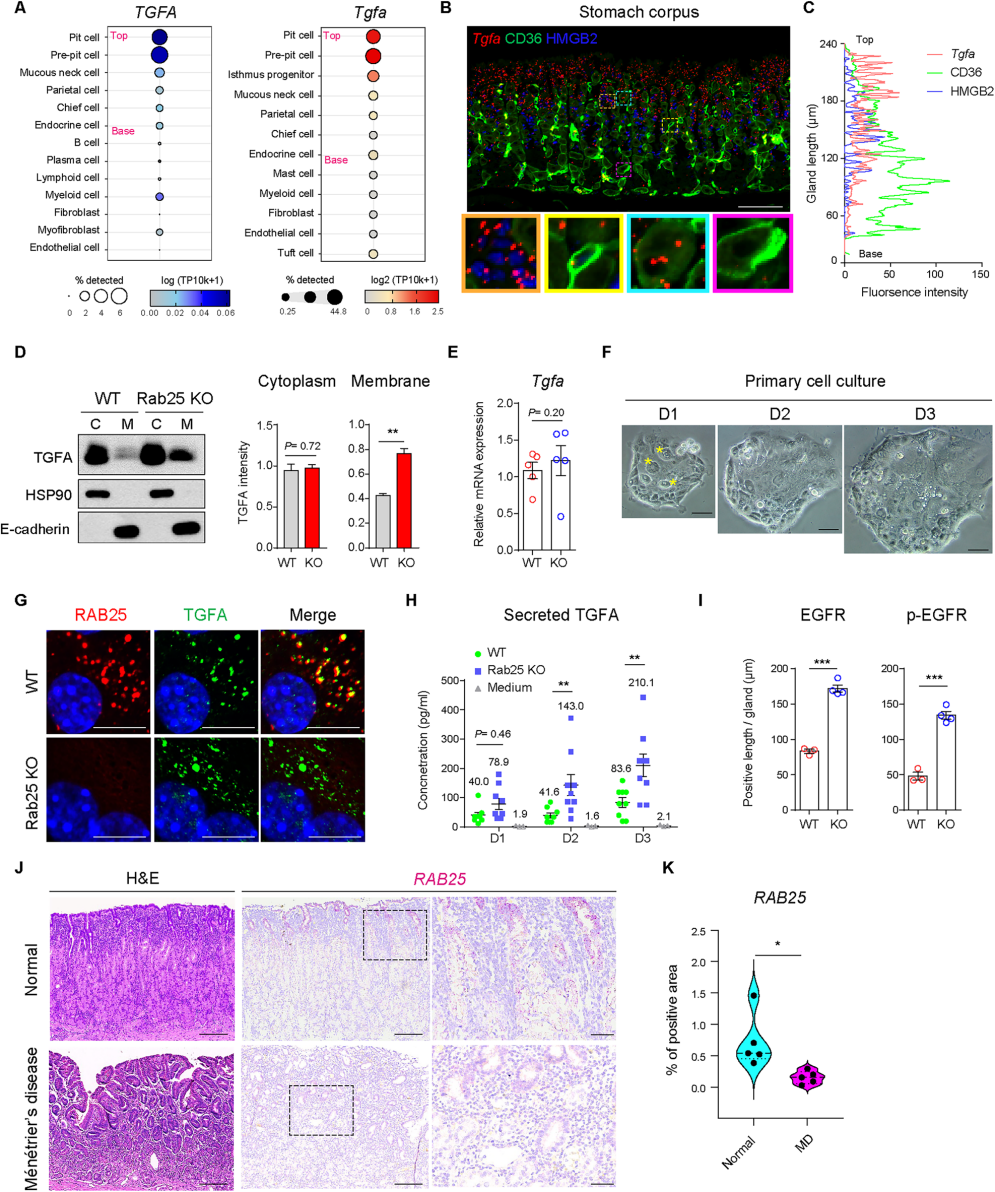
Rab25 deficiency promotes TGFA secretion in gastric epithelial cells. **A** Bubble plot showing variable expression of human *TGFA* and mouse *Tgfa* in defined clusters. Epithelial cells were arranged according to their location (from ‘base’ to ‘top’) within the gland. **B**, **C** Triple immunofluorescence staining for *Tgfa*, CD36, HMGB2 in the corpus of wild-type (WT). Scale bars, 100 μm. Staining intensity (represented as arbitrary units, a.u.) was measured according to gland length. **D** Immunoblot images for TGFA, HSP90 (cytoplasm marker), and E-cadherin (membrane marker) in WT and Rab25 knock-out (KO) mice. Stomach lysate was separated into cytoplasmic fraction ‘C’ and membrane fraction ‘M’. The graph represents the intensity of immunoblot for TGFA in WT and Rab25 KO mice (*n* = 3 per group, two tailed Student’s *t*-test, **P < 0.01). **E** RT-qPCR for *Tgfa* in corpus of WT and Rab25 KO mice (*n* = 5 per group, two tailed Student’s *t*-test). **F** Light microscopes images showing morphological changes in primary cells up to 3 days after seeding. Yellow asterisks indicate matured cells present before primary culture. Scale bars, 100 μm. **G** Immunocytochemistry images for RAB25 and TGFA in the cultured cells derived from WT and Rab25 KO mice. Scale bars, 10 μm. **H** Secreted TGFA level from gastric epithelial cells at day 1 (D1), day 2 (D2) and day 3 (D3) after in vitro culture (*n* = 9 per group, ANOVA multiple comparisons test, **P < 0.01). **I** The graphs represent the positive length (EGFR, p-EGFR) per single corpus gland (*n* = 3–4 per group, two-tailed Student’s *t*-test, ***P < 0.001). **J**, **K** H&E and in situ hybridization images for *RAB25* in the patients with inflamed normal and Ménétrier’s disease. The violin plot indicates the percentage of *RAB25* positive area of the gland region in 10X high-power field (*n* = 5 for each group, two tailed Student’s *t*-test, *P < 0.05). Scale bars, 200 μm (left and middle panels); 50 μm (right panels). All data are represented as mean ± SEM.

Among the diverse EGFR ligands, TGFA appears to be a dominant ligand in corpus homeostasis (Fig. S6A and S6B). Functionally, TGFA promotes lineage commitment of progenitor cells towards pit cells, and this EGFR-binding growth factor is primarily produced in gastric epithelial cells [1, 2, 6]. We sought to define which epithelial cells can produce TGFA. scRNA-seq data revealed that *TGFA* and *Tgfa* were enriched in specific epithelial subsets in human and mice (Fig. 5A). Indeed, *Tgfa* transcripts were mainly expressed in upper corpus cells, including HMGB2+ progenitor cells, HMGB+/CD36+ parietal lineage-committed cells, HMGB-/CD36+ parietal cells, and pit lineage cells (Fig. 5A-C). However, epithelial cells at the base of the gland, where older parietal cells, chief cells, and endocrine cells reside, showed less *Tgfa* expression (Fig. 5A, C). Overall, the *Tgfa* distribution was similar to *Rab25* expression. Although the growth factor was expressed by a relatively broad range of epithelial cells, its receptor EGFR was confined to pit lineage-committed cells in both WT and Rab25 KO mice (Fig. S6C). Those results suggest that a unique TGFA/EGFR axis may accelerate pit cell commitment.

Supporting increased pit cell area in Rab25 KO mice, membrane-transported pro-TGFA was significantly higher in Rab25 KO mice than in WT mice (Figs. 5D, S6D). However, expression of *Tgfa* mRNA and intracellular protein did not differ between WT and Rab25 KO mice (Figs. 5D, E, S6A). This finding suggested that post-translational mechanisms influence the increased pro-TGFA in the membrane fraction. However, the expression of ADAM17, which cleaves pro-TGFA into its secreted form [35], did not differ between WT and Rab25 KO mice (Fig. S6E). Since pro-TGFA is cleaved and secreted from the cell membrane, we cultured primary gastric epithelial cells to investigate whether the secretion of TGFA is linked to increased membrane TGFA. Sequential microscopic images showed that the newly matured cells were derived from progenitor cells, forming an enlarged colony (Fig. 5F). Prior to further experiments, we verified that an equal number of glands from WT and KO mice could generate a similar number of mature cells (Fig. S6F, G). Of note, the punctate expression of RAB25 was recapitulated in vitro in epithelial cells, but the expression disappeared in Rab25 KO cells (Fig. S6H). Additionally, perinuclear RAB25 was closely associated with TGFA+ vesicles (Fig. 5G). Most importantly, during the maturation phase, secreted TGFA was gradually and significantly increased in Rab25 KO-derived epithelial cells compared to WT-derived epithelial cells (Fig. 5H). As the secretion of TGFA was promoted, the positive area of EGFR and phospho-EGFR staining expanded in Rab25 KO mice compared to WT mice (Figs. 5I, S6I).

### TGFA neutralization can ameliorate hyperplastic lesions in Rab25 KO mice

**Fig. 6:**
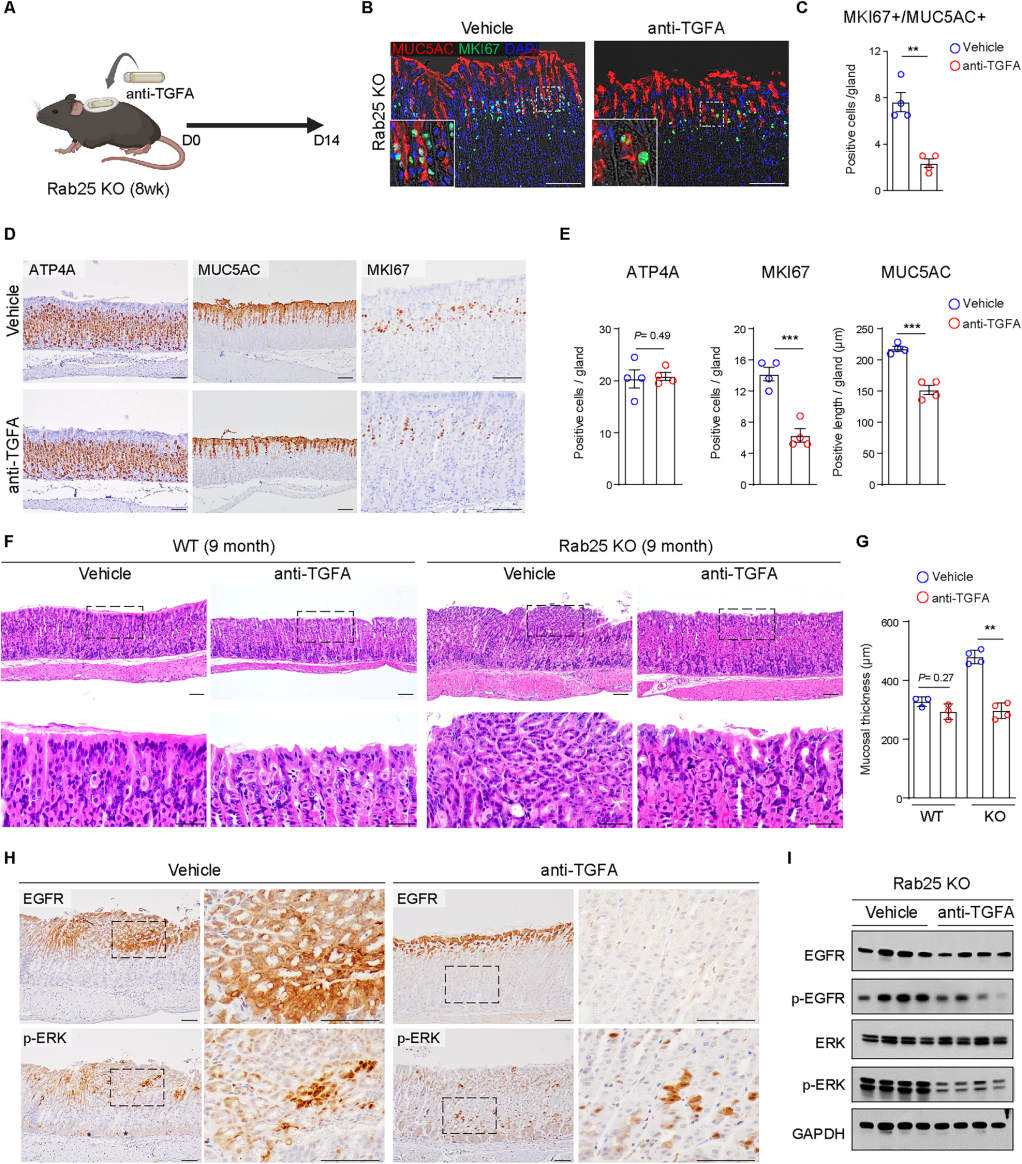
Therapeutic effects of treatment of TGFA antibody in Rab25 knock-out (KO) mice. **A** Schematic image showing implantation of an osmotic pump containing anti-TGFA (5 mg/kg) to provide antibodies continuously for 14 days in Rab25 KO mice. **B**, **C** Immunofluorescence images for MUC5AC and MKI67 in corpus of vehicle- and TGFA antibody-treated Rab25 KO mice. Scale bars, 100 μm. The graph represents the number of MKI67 + /MUC5AC+ cells in a corpus gland (n = 4 per group, two-tailed Student’s *t*-test, **P < 0.01). **D**, **E** Immunohistochemistry images for ATP4A, MUC5AC, MKI67. Scale bars, 100 μm. The graphs represent the number of positive cells (ATP4A and MKI67) and positive length (MUC5AC) per single corpus gland (*n* = 4 per group, two-tailed Student’s *t*-test, ***P < 0.001). **F** Histopathological observation of vehicle- and TGFA antibody-treated 9-month-old WT and Rab25 KO mice. Scale bars, 100 μm (upper panels); 50 μm (lower panels). **G** The graph represents the mucosal thickness (*n* = 3–4 per group, two tailed Student’s *t*-test, **P < 0.01). **H** Immunohistochemistry images for EGFR and p-EGFR in corpus of vehicle- and TGFA antibody-treated 9-month-old Rab25 KO mice. Scale bars, 50 μm. **I** Immunoblot images for EGFR, p-EGFR, ERK, p-ERK in the corpus of vehicle- and TGFA antibody-treated 9-month-old Rab25 KO mice. GAPDH was used as la oading control. All data are represented as mean ± SEM.

Overexpression of TGFA can cause a Ménétrier’s disease-like phenotype characterized by massive foveolar hyperplasia in mice [16]. We assessed *RAB25* expression levels in a patient with Ménétrier’s disease characterized by massive foveolar hyperplasia (Fig. 5J). The result showed that the *RAB25* expression was lower in Ménétrier’s disease than in inflamed normal (Fig. 5J, K). Of note, we demonstrated that Rab25 deficiency leads to excessive secretion of TGFA from gastric epithelial cells and causes foveolar hyperplasia. Thus, we implanted osmotic pumps containing anti-TGFA to block the TGFA-mediated pathway and then evaluated the relevant phenotypes in Rab25 KO mice (Fig. 6A). In 8-week-old Rab25 KO mice, neutralization of TGFA suppressed the increase in pre-pit cells (Fig. 6B, C). The antibody treatment reduced not only cell proliferation, but also foveolar hyperplasia (Fig. 6D, E). In contrast, we did not see any changes in parietal cells (Fig. 6D, E). To verify whether neutralization of TGFA can prevent gastric disease in KO mice, we also implanted the osmotic pumps in 9-month-old Rab25 KO mice. Importantly, treatment of TGFA antibody dramatically reduced mucosal thickness in the older Rab25 KO mice compared to untreated control mice and ameliorated hyperplastic lesions (Fig. 6F, G). The antibody treatment did not cause significant changes in WT mice (Fig. 6F, G). Furthermore, expansion of EGFR+ and phospho-ERK+ cells in Rab25 KO mice was suppressed in antibody-treated mice (Fig. 6H, I). Most importantly, the suppression was prominent in gastric pit regions, suggesting that constitutive activation of TGFA/EGFR signaling in gastric pit regions contributes to the induction of hyperplastic lesions (Fig. 6I).

## Discussion

Since Rab25 plays crucial roles in intracellular trafficking pathways, a number of reports have shed light on its target-associated molecular mechanisms. In both the intestine and skin, Rab25, present in recycling endosomes, governs integrin distribution in epithelial cells, helping the maintenance of cell polarity [27, 28]. Rab25 also contributes to skin barrier function by coordinating maturation of the filaggrin granule, which is a unique compartment of granular keratinocytes [26]. Additionally, Rab25 can collaborate with CLIC3 and lead to retrograde transportation of integrins from lysosomes to the cell membrane [36]. Notably, similar to our result, a previous report demonstrated that loss of Rab25 facilitates secretion of vascular endothelial growth factor [37]. Collectively, it is likely that Rab25 has tissue-specific function by mediating a broad range of trafficking pathways for its target molecules, and these molecular/physiological roles of Rab25 are associated with the progression of diverse human diseases [25, 27, 28].

In gastric epithelial cells, Rab25 appears to be involved in controlling the secretory pathway of a growth factor that promotes pit cell lineage commitment in homeostatic conditions. RAB25 was closely distributed with TGFA+ vesicles in the perinuclear region, implying that it may regulate TGFA exocytosis and thereby suppress excessive secretion of growth factors from epithelial cells. Indeed, TGFA released after cleavage at the cell surface was increased in Rab25 KO mice compared to WT mice. Although our data show increased membrane-bound and secreted TGFA upon Rab25 loss, the exact trafficking step regulated by Rab25 remains unresolved. Our results indicate that ADAM17-mediated cleavage is unlikely to account for the phenotype, suggesting that Rab25 may affect alternative steps such as vesicle retention or exocytosis. Consequently, the lack of Rab25 resulted in foveolar hyperplasia due to hypersecretion of TGFA. Furthermore, long-term exposure to TGFA/EGFR signaling in Rab25 KO mice can induce gastric disease. Although multiple factors, such as hypergastrinemia and achlorhydria, have also been suggested to promote foveolar hyperplasia [8–10], there were no significant changes in gastric pH and gastrin level in Rab25-deficient mice. This indicates that TGFA directly impacts the EGFR+ pit cells.

The main producer of TGFA was still unclear in the stomach despite a variety of previous reports. Traditional techniques in molecular biology have indicated that TGFA exists in vesicle compartments in parietal cells, but not in chief cells [6, 15, 38]. Recent single-cell sequencing using mouse gastric tissues demonstrated that pit cells and progenitor cells also have *Tgfa* transcripts [1, 2]. Here, we provided evidence for the localization of TGFA in the corpus. *Tgfa* was enriched in progenitor cells, pit cells, and pre-parietal cells, and some committed parietal cells. Overall, as parietal cells migrate from the progenitor zone to the base of the gland and fully differentiate, they appear to lose their *Tgfa* transcripts (Fig. 5A-C). Typically, pit cells can survive 5 days, but parietal cells and chief cells can survive over 100 days. Taken together, the undifferentiated progenitor cells and relatively young parietal cells in the pit region are likely to be stable suppliers of TGFA. Thus, they can continuously lead to normal pit cell commitment by providing a growth factor to progenitors despite early removal of TGFA+ cells in top of the gland.

Takada et al., [1] recently showed that TGFA treatment in gastric organoids accelerated pit cell maturation via EGFR/ERK activation. However, they suggested that proliferation was independent of the growth factor, or rather suppressed organoid growth. These results on proliferation are in contrast with in vivo findings of Rab25 KO mice. In our recent paper using scRNA-seq, we demonstrated that EGFR expression is especially prominent in a proliferative progenitor and pre-pit cluster. [2] Given that Rab25 KO mice contain many short-lived pit cells, it can be expected that the dividing cells supported by EGFR signaling would be much higher in Rab25 KO mice than in WT mice. Furthermore, increased proliferation is also a common action of the TGFA/EGFR axis, which promotes hyperplasia in diverse organs [39, 40]. The possibility that organoid culture does not perfectly mimic in vivo conditions also cannot be ruled out.

TGFA/EGFR signaling is not only important for maintaining pit homeostasis but also appears to be responsible for the progression of human gastric disease. Ménétrier’s disease is a rare gastric lesion characterized by upregulation of TGFA/EGFR, giant rugal folds, and massive foveolar hyperplasia [39, 41]. We previously established metallothionein (MT)-TGFA mice overexpressing TGFA and found that the mice can recapitulate the pathological features of Ménétrier’s disease [17]. Notably, MT-TGFA mice did not show prominent changes in the antrum, similar to Rab25 KO mice. In a previous clinical report, antibody neutralization of EGFR in a patient with Ménétrier’s disease ameliorated disease severity [18]. Those results indicate that TGFA-induced EGFR activation is a crucial factor in the development of Ménétrier’s disease. In present study, expanded gastric rugal folds containing foveolar hyperplasia were observed in aged Rab25 KO mice. In addition, treatment with anti-TGFA antibody prevented the corpus lesion along with attenuation of EGFR and its downstream signaling. Overall, the pathological phenotype seen in Rab25 KO mice resembles Ménétrier’s disease. Nevertheless, a limitation in the present study is that it was not possible to show the direct evidence connecting loss of Rab25 and progression of Ménétrier’s disease [39]. We also acknowledge that the human data presented here are limited and should be regarded as preliminary, warranting further validation in larger patient cohorts.

Taken together, Rab25 loss can promote pit cell commitment by enhancing TGFA secretion in gastric epithelial cells, and excessive TGFA secretion due to loss of Rab25 can cause EGFR activation and lead to a stomach phenotype resembling human Ménétrier’s disease.

## Materials and Methods

### Mice

All mice were bred under specific pathogen-free conditions in the animal facility at Yonsei University College of Medicine. Male WT and *Rab25* KO mice with a 129J background were used for all experiments; the KO mice were generated and genotyped according to a previous study [26]. Mice were randomly assigned to experimental and control groups, and the main investigator was blinded to group allocation during outcome assessment. For neutralizing TGA in vivo, osmotic pumps containing anti-TGFA (5 mg/kg) were implanted into Rab25 KO mice, and the antibodies were constitutively administered for 14 days before sacrifice.

### In situ hybridization

For in situ hybridization, human and mouse slides were incubated in a baking oven at 60°C for 1 h and cooled at room temperature for at least 30 mins. In situ hybridization was performed with the RNAscope detection kit (Advanced Cell Diagnostics, Newark, CA, USA) according to the manufacturer’s protocol. In brief, deparaffinized formalin-fixed paraffin sections were pretreated with hydrogen peroxide, target retrieval solution, and ready-to-use protease. The slides were incubated with human *RAB25*- or mouse *Rab25*- or mouse *Tgfa*-specific probe in a humidity chamber at 40 °C for 2 h. For detection, 6 sequential steps of signal amplification were conducted before developing the slides using Fast Red and fluorophore reagent. The positive intensity was measured using QuPath-0.4.3 software.

### Quantitative reverse transcription polymerase chain reaction (RT-qPCR)

For RNA extraction, corpus samples dipped in RNAlater were transferred to 1.5-mL conical tubes containing TRIzol solution (Invitrogen, Waltham, MA, USA). The tissue samples were then completely homogenized using a tissue homogenizer. RNA derived from tissues was extracted using TRIzol reagent according to the manufacturer’s protocol. A total of 1 µg of RNA was synthesized into cDNA with a reverse transcription system (Promega, Madison, WI, USA), and qPCR was conducted using SYBRgreen (Takara, Kusatsu, Japan) under the following reaction protocol: 95 °C for 3 min, 40 cycles of 95 °C for 10 s and 60 °C for 30 s. The mouse primer sets used for RT-qPCR are described below.

m*Rab25*; FW: CTTAAAAGCTGAGAGTTG, RV: CTCGCCGATCAGCACCAC.

m*Tgfa*; FW: AGCATGTGTCTGCCACTCTG, RV: TGGATCAGCACACAGGTGAT.

m*Areg*; FW: CATTATGCAGCTGCTTTGGA, RV: GTCGTAGTCCCCTGTGGAGA.

m*Egf*; FW: ACGCCGAAGACTTATCCAGA, RV: CATGCTGCCTTGAAGACGTA.

m*Ereg*; FW: CAGGCAGTTATCAGCACAAC, RV: CCTTGTCCGTAACTTGATGG.

m*Gapdh*; FW: AACAGCAACTCCCACTCTT, RV: CCTGTTGCTGTAGCCGTATT.

### Physiological and histopathological analysis

After the mice were sacrificed, blood was collected and transferred to a blood collection tube (Greiner, Kremsmünster, Austria), followed by centrifugation at 10 000 rpm for 10 min at 4 °C to obtain serum. Measurements of gastrin were carried out by GC Cell, Inc. (Korea). After blood collection, stomach tissues were cut along the greater curvature and gastric pH was measured using pH paper (Fisher Scientific, Waltham, MA, USA). For histopathological analysis, stomach specimens were fixed in 4% paraformaldehyde for 24 h with gentle shaking and embedded in paraffin. Then, paraffin sections were deparaffinized and rehydrated in xylene and alcohol. Afterwards, slides were dipped in 0.1% Mayer’s hematoxylin for 10 min and then in 0.5% eosin. After staining, the following sequential washing steps were performed: distilled water until the eosin stopped streaking, dip in 50% ethanol 10 times, dip in 70% ethanol 10 times, 95% ethanol for 30 s, and 100% ethanol for 1 min. The sections were covered with mount solution (Thermo, Waltham, MA, USA), and the pathological diagnoses were determined by an experienced mouse pathologist (Ki Taek Nam, DVM).

### Immunohistochemistry

For immunostaining, 4-µm paraffin sections were dipped in xylene three times and rehydrated in a descending alcohol gradient (100% twice, 95% twice, and 70% once). Antigen retrieval (Agilent) was then performed for 15 min using a pressure cooker, and the slide chamber was cooled on an ice bucket for at least 1 h. The slides were incubated in 3% H_2_O_2_ for 30 min to block endogenous peroxidase. Slides were washed twice with phosphate-buffered saline (PBS) and incubated with blocking solution (Agilent, Santa Clara, CA, USA) for 2 h at room temperature. Before 1 h of protein blocking, slides were pre-treated with M.O.M. reagent (Vector Laboratories, Newark, CA, USA) for 2 h when the primary antibody was of mouse origin. The sections were incubated with the primary antibodies overnight at 4°C. After three washes in PBS, the sections were incubated in the horseradish peroxidase-conjugated secondary antibody (Agilent) for 15 min at room temperature. DAB substrate (Agilent) was used for signal development, and Mayer’s hematoxylin was used for counterstaining. The positivity was measured using QuPath-0.4.3 software. For immunofluorescence staining, primary antibodies were detected with anti-Cy2-, anti-Cy3-, and anti-Cy5-conjugated secondary antibodies. Immunofluorescence images were taken with an EVOS-FL fluorescence microscope and an LSM800 confocal microscope.

### Western blotting

For primary cells, cells were harvested and incubated in protein lysis buffer (20 mM Tris [pH 7.4], 0.15 M NaCl, 2.5 mM EDTA, 1% Triton X-100) for 40 minutes in an ice bucket. For mice, the same region of the corpus was collected using a biopsy punch and the specimens into 1.5 ml microtubes. Using a tissue grinder, the stomach was homogenized and then lysed with protein lysis buffer (20 mM HEPES [pH 7.0], 0.15 M NaCl, 10% Glycerol, 1% Triton X-100, 1 mM EDTA, 1 mM EGTA, 10 mM β-phosphoglycerate) containing protease and phosphatase inhibitor cocktails (Thermo). For isolation of membrane and cytoplasm fractions, we used Mem-PER™ Plus Membrane Protein Extraction Kit (Thermo). All protein lysates were collected by centrifugation (13 000 rpm, 15 min) and boiled in 1X SDS-PAGE sample buffer (62.5 mM Tris–HCl [pH 6.8], 2% SDS, 5% β-mercaptoethanol, 10% glycerol, 0.01% bromophenol blue) after measurement of protein concentrations using BSA buffer. Then, 20 μg of protein sample was separated by SDS-PAGE and transferred to a PVDF membrane (Millipore, Burlington, MA, USA). The membranes were incubated overnight at 4°C with primary antibodies with gentle shaking. After primary antibody incubation, HRP-conjugated secondary antibodies (Jackson Laboratories, Bar Harbor, ME, USA) were incubated for 1 hour at room temperature with gentle shaking. ECL substrate (Visual protein, Taipei, Taiwan) was used for signal detection.

### Transmission electron microscopy

The corpus samples were cut into 1×1-mm sections using a blade and then fixed for 24 h in 2% glutaraldehyde–paraformaldehyde in 0.1 M PBS, followed by washing in 0.1 M phosphate buffer. The samples were then post-fixed with 1% OsO_4_ dissolved in phosphate buffer for 2 h, dehydrated in an ascending graded series (50–100%) of ethanol, and infiltrated with propylene oxide. Specimens were embedded using the Poly/Bed 812 kit (Polysciences). After pure fresh resin embedding and polymerization at 65°C in a vacuum oven (DOSAKA) for 24 h, 200–250 nm sections were initially cut and stained with toluidine blue (Sigma, St. Louis, MO, USA) for light microscopy. Sections were cut using a LEICA EM UC-7 with a diamond knife and transferred onto copper and nickel grids. Sections cut at a thickness of 70 nm were double-stained with 6% uranyl acetate for 20 min and with lead citrate for contrast staining. All the sections were examined under a transmission electron microscope (JEOL, Akishima, Japan) at 80 KV.

### Immunocytochemistry

For immunocytochemistry, cells were seeded on growth factor-reduced Matrigel (Corning, New York, USA)-coated 24-well chamber slides and grown and fixed with 4% paraformaldehyde for 15 min at room temperature and washed with ice-cold PBS three times. The cells were then incubated with 0.25% Triton X-100 for 15 min at room temperature for permeabilization, followed by the addition of 1% bovine serum albumin for 30 min at room temperature for blocking. After three washes in ice-cold PBS between each step, the cells were incubated with the primary antibodies for 2 h at room temperature. After two washes in ice-cold PBS, the slides were incubated with Cy3-conjugated anti-rabbit IgG and Cy5-conjugated anti-mouse IgG secondary antibodies for 1 h at room temperature in the dark. Counterstaining of the nucleus was performed using DAPI (Sigma). Confocal images were captured using a Zeiss LSM800 microscope.

### Primary cell culture

For gastric epithelial cell culture, the mice stomach was opened along the greater curvature, and residual food was removed by PBS washing. The antrum and forestomach were removed. Then, the corpus was minced with scissors, and the tissues were then transferred to a digestion solution (Minimum Essential Medium containing 20 mM HEPES [pH 7.0], 0.2% BSA, 0.1 mg/ml Gentamycin, 3 mg/ml Collagenase type I) and incubated at 37°C for 30 min with rotation at 40 rpm. After centrifugation at 50 g for 8 min, the tissue pellet was transferred to a lid of 60 mm culture plate through a 1000 P wide-bore tip and gently compressed using a cover slide to isolate the gland. After squeezing, all isolated cells were washed and collected into a 15 ml tube using 4 ml washing medium (DMEM/F12K containing 0.1 mg/ml Gentamycin, 0.5 mM DL -Dithiothreitol). The resuspended pellet was filtered through a 100 μm strainer, followed by another centrifugation step at 200 g for 5 min. The media was aspirated, and the pellet was resuspended in 1 ml washing medium and transferred into a 1.5 ml tube. After another centrifugation, the glands containing epithelial cells were resuspended with the established cell medium (DMEM/F12K containing 0.2% BSA, 0.1 mg/ml Gentamycin, 1X Penicillin-Streptomycin, 1X Insulin-Transferrin-Selenium, 1 μM Hydrocortisone, 100 ng/ml EGF), and 200 glands were seeded on a collagen-coated plate. For in vitro parietal cell activation, 100 µM Histamine (Sigma) and 20 µM IBMX (Sigma) were supplemented.

### Antibodies

The following primary antibodies were commercially purchased and used: anti-MUC5AC (Thermo, #MA5-12178), anti-CD44v9 (Cosmo bio, Kyoto, Japan, # LKG-M002), anti-MKI67 (Abcam, Cambridge, UK, #ab16667), anti-MIST1 (Cell signaling technology, Danvers, MA, USA, #14896), anti-ATP4A (MBL, Japan, #DO31-3), GSII (Thermo, #L21415), anti-CHGA (Abcam, #15160), anti-RAB25 (Thermo, #MA5-15587), anti-EZRIN (Cell signaling technology, #3145), anti-HSP90 (Abcam, #ab13492), anti-E-cadherin (Santacruz, Dallas, TX, USA, #sc-59778), anti-TGFA (Novus, #NBP2-34296), anti-GAPDH (Abcam, #ab9485), anti-EGFR (Abcam, #ab52894), anti-pEGFR (Abcam, #ab5644), anti-ERK1/2 (Cell signaling technology, #4695), anti-pERK1/2 (Cell signaling technology, #4370), anti-GAST (Zymed, Oxnard, CA, USA, #18-0062), anti-CD36 (R&D Systems, Minneapolis, MN, USA, #AF2519), anti-HMGB2 (Abcam, #ab124670), anti-ADAM17 (Invitrogen, #PA5-27395).

### Bioinformatics of human samples

For scRNA-seq analysis for inflamed normal samples obtained from non-cancerous adjacent site, we utilized our scRNA-seq data (GSE150290) and re-clustered three inflamed normal patients [30]. Briefly, a gene count matrix was generated from the raw fastq files mapped to the GRCh38 reference genome using Cell Ranger software (v7.0.0) supplied by 10× Genomics. In accordance with the previous strategy, we filtered out low-quality cells based on the following criteria: RNA counts greater than 20,000; RNA feature counts less than 200 or greater than 2000; percentage of mitochondrial genes and hemoglobin genes upper than 30% and 10%, respectively. The remaining cells were normalized for each sample separately and then integrated using the harmony method implemented in the Seurat R package (v5.0.0). Subsequently, unsupervised cell clusters were determined using the FindClusters function (resolution = 0.3), and the clusters were annotated by manual inspection of differentially expressed genes compared to known cell type markers. For scRNA-seq analysis for mouse corpus samples, we utilized published scRNA-seq data [31] and re-clustered raw data from WT mice. A gene count matrix was aligned and generated from the raw fastq files mapped to the GRCm39 reference genome using the Cell Ranger software. We filtered out low-quality cells based on the following criteria: cells ranked in the top 1% of UMI counts; RNA feature counts less than 200 or greater than 5800; percentage of mitochondrial genes greater than 25%. Normalization of the aligned data, unsupervised cell clustering, and subsequent analysis were conducted using Partek Flow (Partek).

### Statistical analysis

Statistical analyses were performed using GraphPad Prism software v 9.0. Statistical significance was determined using the unpaired Student *t*-test or ANOVA analysis of variance with Sidak’s multiple comparison test. Animal and in vitro experiments were validated through repeated trials, and for significance analysis, a minimum of *n* = 3 samples per group was used. All data are presented as the mean ± standard error of the mean. *P* < 0.05 was considered statistically significant. In Partek Flow’s Hurdle model, significance (*P*-value) is obtained through a two-step process: first, a binomial test determines if genes are expressed or not between groups (zero vs. non-zero counts), followed by a count-based test (e.g., negative binomial regression) for non-zero counts to compare expression levels. The *P*-values from these two steps are combined to provide an overall significance for differential expression.

### Study approval

All animal experiments were conducted in accordance with the Public Health Service Policy in Humane Care and Use of Laboratory Animals and were approved by the IACUC (2021-0174) of the Department of Laboratory Animal Resources of Yonsei University College of Medicine, an AAALAC-accredited unit (#001071). The gastric specimens from patients with Ménétrier’s disease were available from our previous study [12].

## Supplementary information

Supplemental Material

## Data Availability

All data that support the findings of this study can be found in the manuscript, figures, and supplementary data or are available from the corresponding authors upon request.
