# Supplementary material for: RAB25 modulates pit cell commitment by coordinating transforming growth factor-alpha secretion from gastric epithelial cells
Source: Cell Death Dis. 2025 Dec 9;17(1):57. doi: 10.1038/s41419-025-08316-2 (PMC12824383; doi:10.1038/s41419-025-08316-2)
Supplement: Supplementary file 1 — Supplemental Material [file 41419_2025_8316_MOESM1_ESM.pdf]

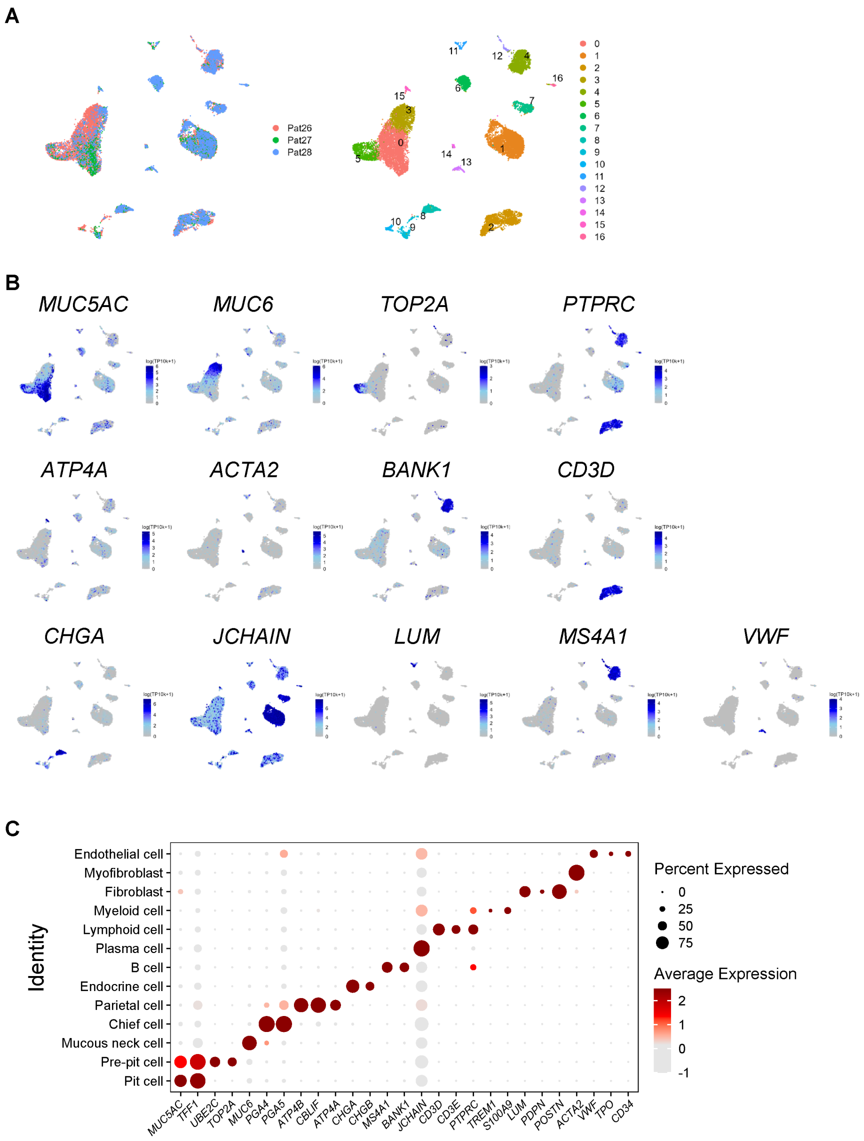

Fig S1. (A) Uniform manifold approximation and projection (UMAP) showing the distribution of all cell clusters in three different human inflamed normal patients. Each color in UMAP represents individual patient-derived cells (left) and subpopulations of total single cells (right). **(B-C)** UMAP and bubble plot showing the expression of marker genes of each cell type.

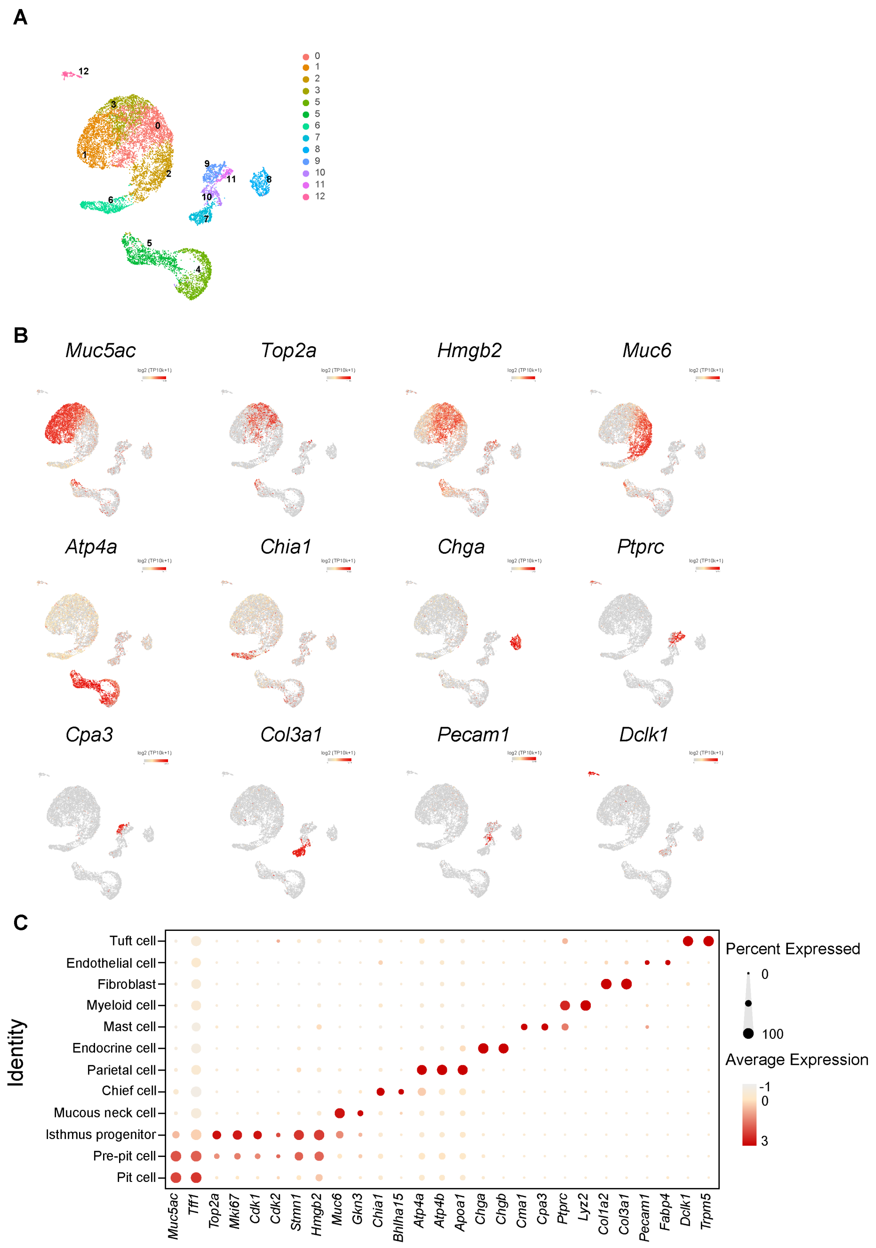

Fig S2. (A) Uniform manifold approximation and projection (UMAP) showing the distribution of all cell clusters in corpus of wild-type (WT) mice. Each color in UMAP represents individual subpopulations of total single cells. **(B-C)** UMAP and bubble plot showing the expression of marker genes of each cell type.

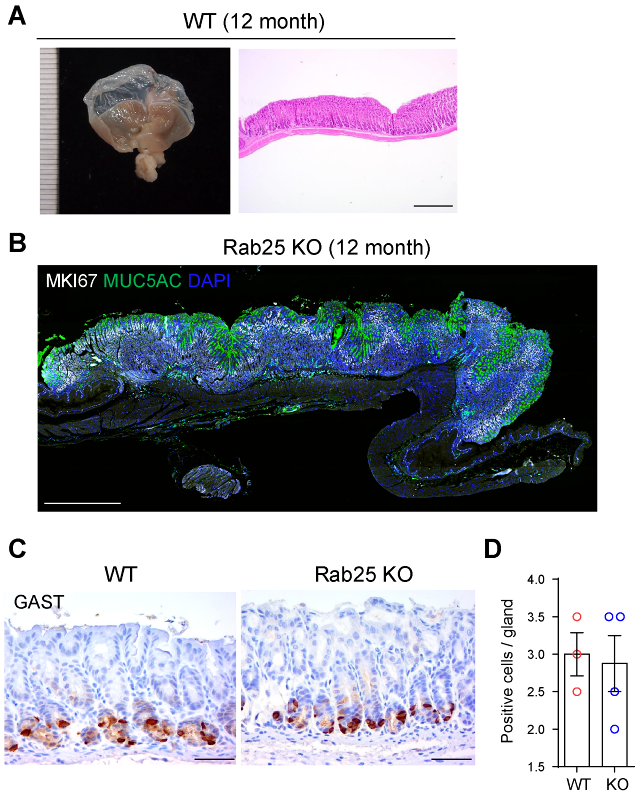

Fig S3. (A) Macroscopic and histopathological observation of 12-month-old wild-type (WT) mice. Scale bars, 500 μm. **(B)** Immunofluorescence images for MUC5AC and MKI67 in 12-month-old Rab25 knock-out (KO) mice. Scale bar, 1 mm. **(C-D)** Immunohistochemistry for GAST of antrum of 1 month old WT and Rab25 KO mice. Scale bars, 100 μm. The graphs represent the number of GAST+ cells per single antrum gland (n= 3-4 per group). All data are represented as mean ± SEM.

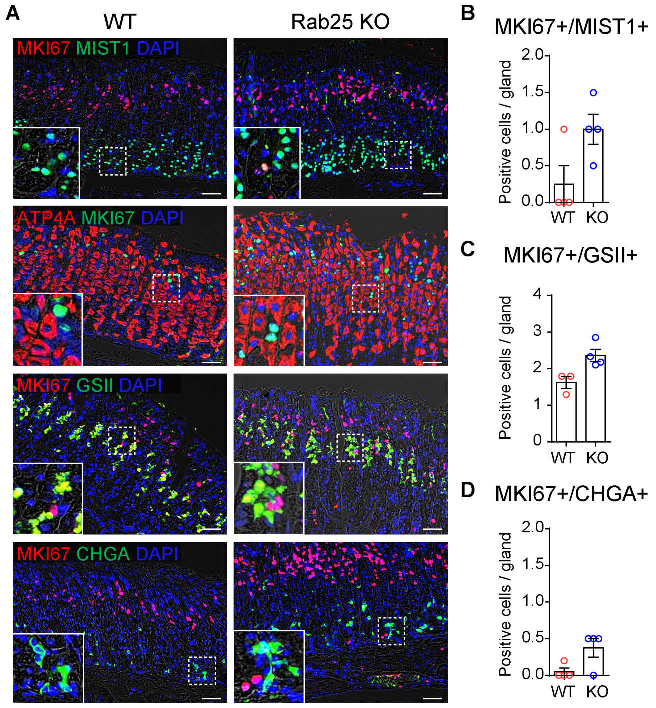

Fig S4. (A-D) Immunofluorescence images for MIK67 and other lineage markers of corpus (MIST1, ATP4A, GSII and CHGA) of 1 month old wild-type (WT) and Rab25 knock-out (KO) mice. Scale bars, 50 µm. The graphs represent the number of positive cells per single corpus gland (n= 3-4 per group, two tailed Student's *t*-test). All data are represented as mean ± SEM.

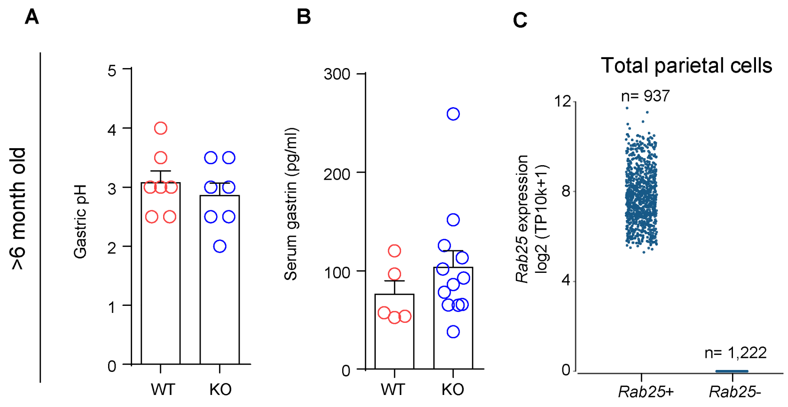

Fig S5. (A-B) The graphs represent the measurement on gastric pH and serum gastrin in aged wild-type (WT) and Rab25 knock-out (KO) mice. **(C)** The graph represents the normalized expression levels of *Rab25* expressed in a cluster of parietal cells. The cells were divided into positive cells and negative cells depending on the presence of absence of *Rab25* (n = 937 and 1,222 for each group). All data are represented as mean ± SEM.

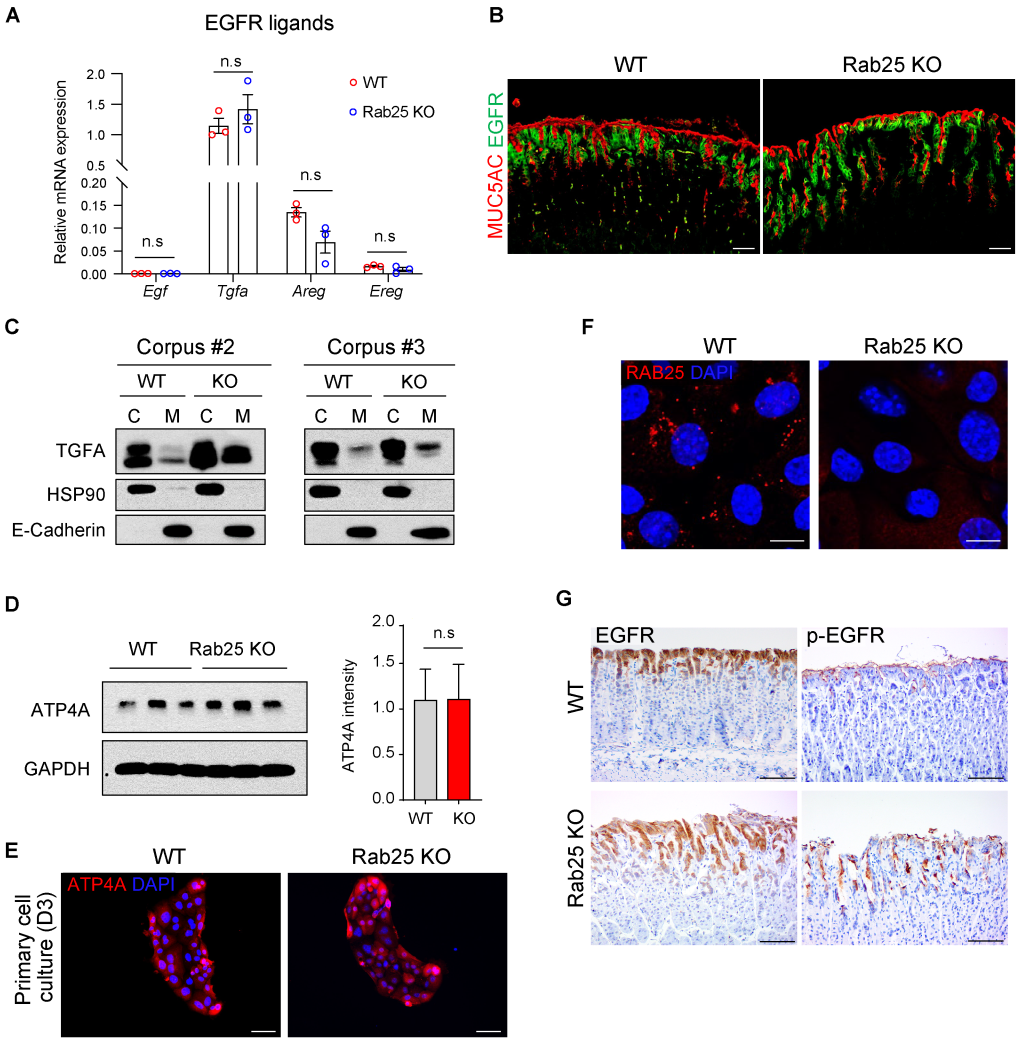

Fig S6. (A) RT-qPCR for EGFR ligands in corpus of wild-type (WT) and Rab25 knock-out (KO) mice (n= 3 per group, two tailed Student's *t*-test). **(B)** Immunofluorescence images for MUC5AC and EGFR in corpus of WT and Rab25 KO mice. Scale bars, 50 µm. **(C)** Immunoblot images for TGFA, HSP90 (cytoplasm marker) and E-cadherin (membrane marker) in two different corpus samples from WT and Rab25 KO mice. **(D)** Immunoblot images for ATP4A and GAPDH in cultured cells derived from WT and Rab25 KO mice at day3. GAPDH was used as loading control. The graph represents the intensity of immunoblot for ATP4A (n= 3 per group, two tailed Student's *t*-test). **(E-F)** Immunocytochemistry image for ATP4A and RAB25 in the cultured cells at day3. Scale bars, 50 µm. **(G)** Immunohistochemistry images for

EGFR and p-EGFR in corpus of WT and Rab25 KO mice. Scale bars, 100 μm. All data are represented as mean ± SEM.
